# Theoretical Study on the Allosteric Regulation of an Oligomeric Protease from *Pyrococcus horikoshii* by Cl^−^ Ion

**DOI:** 10.3390/molecules19021828

**Published:** 2014-02-07

**Authors:** Dongling Zhan, Jiao Sun, Yan Feng, Weiwei Han

**Affiliations:** 1 Key Laboratory for Molecular Enzymology and Engineering of Ministry of Education, Jilin University, Changchun 130023, China; E-Mail: zdlgale@126.com; 2 College of Food Science and Engineering, Jilin Agricultural University, Changchun 130118, China; 3 Norman Bethune College of Medicine, Jilin University, Changchun 130021, China; E-Mail: jiaosun@jlu.edu.cn

**Keywords:** oligomeric protease, allosteric regulation, molecular dynamics simulation

## Abstract

The thermophilic intracellular protease (PH1704) from *Pyrococcus horikoshii* that functions as an oligomer (hexamer or higher forms) has proteolytic activity and remarkable stability. PH1704 is classified as a member of the C56 family of peptidases. This study is the first to observe that the use of Cl^−^ as an allosteric inhibitor causes appreciable changes in the catalytic activity of the protease. Theoretical methods were used for further study. Quantum mechanical calculations indicated the binding mode of Cl^−^ with Arg113. A molecular dynamics simulation explained how Cl^−^ stabilized distinct contact species and how it controls the enzyme activity. The new structural insights obtained from this study are expected to stimulate further biochemical studies on the structures and mechanisms of allosteric proteases. It is clear that the discovery of new allosteric sites of the C56 family of peptidases may generate opportunities for pharmaceutical development and increases our understanding of the basic biological processes of this peptidase family.

## 1. Introduction

The thermophilic intracellular protease from *Pyrococcus horikoshii* (PH1704) that functions as an oligomer (hexamer or higher forms) has proteolytic activity and remarkable stability [1,2]. PH1704 is classified as a member of the C56 family of peptidases using the MEROPS search tool [3]. This family contains *Pyrococcus furiosus* intracellular protease I (PfpI) [4,5], PH1704 [1,2], *Bacillus subtilis* general stress protein 18 (GSP18) [6], protein YhbO [7,8], DR1199 from *Deinococcus radiodurans* [9] and so on. Among this family, DR1199 and YhbO are classified by the MEROPS peptidase database as non-peptidase homologs of the C56 family of peptidases.

**Figure 1:**
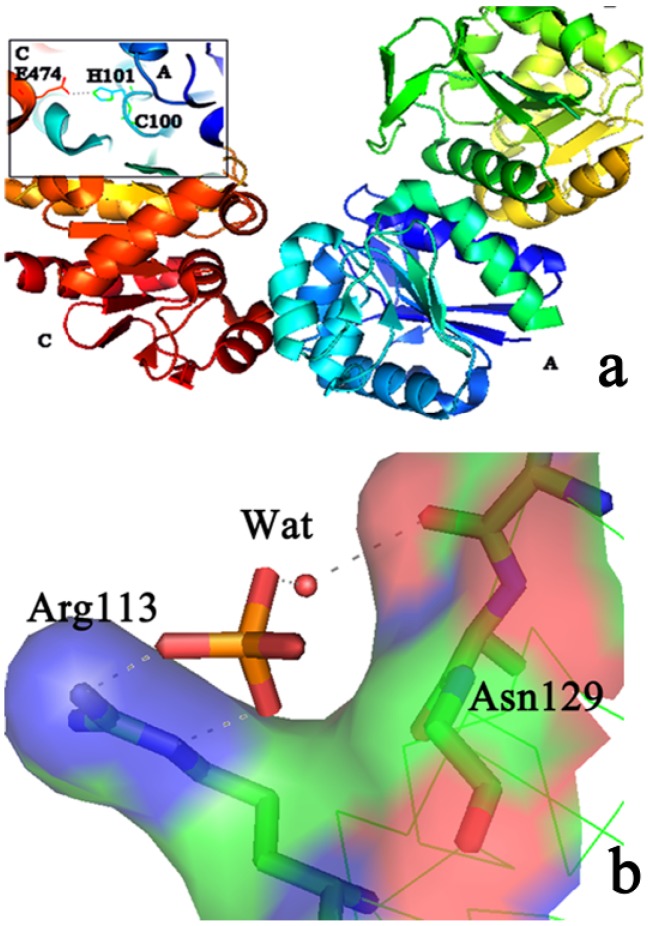
(**a**) The catalytic triad Cys100, His101 and Glu474; (**b**) The binding mode of SO_4_^2−^ in PH1704.

PH1704 shares 90% sequence identity with PfpI, the most well characterized protein in this family, which is an intracellular cysteine peptidase characterized by its stability and the speculated proteolytic activity of the thermophilic archaebacterium. The 3D structure of PH1704 shows an α/β-sandwich fold, containing a similar domain characterized by a sharp turn between the *β*-strand and the α-helix. The fold resembles that of class I glutamine amidotransferase (GATase) [10], which is characterized by a conserved Cys–His–Glu active site. PH1704 forms a hexameric structure, and the active sites are formed at the interfaces between three pairs of monomers. The shared active site between subunits A and C of PH1704 performs proteolytic cleavage through a Cys–His–Glu catalytic triad: Cys100 and His101 residue on the A subunit, and Glu474 on the neighboring C subunit (seen in Figure 1a).

Allosteric regulation of the function of a protein occurs when a small activator or inhibitor molecule binds away from the oligomeric protein’s normal active site [11]. In recent years, several oligomeric proteases have been found to possess allosteric sites, and binding of small molecules to these sites could result in the modulation of enzyme activities [12,13,14]. For example, a novel allosteric site in protein tyrosine phosphatase 1B (PTP1B) was discovered to bind small molecules allowing an opportunity to avoid troubles associated with inhibitors of the catalytic site. Allosteric inhibition in PTP1B is a promising, new strategy for treatment of obesity and type II diabetes [15,16]. Another example of the importance of allosteric sites has been discovered with caspases, which are mediators of apoptosis and the inflammatory response [15,17]. Caspases are an important class of drug targets for stroke, ischemia, and cancer [17], but it has been difficult to find drug-like caspase inhibitors because of a strong preference for an acidic side chain and an electrophilic functionality to bind at the active site [17]. However, the binding of various ligands at the allosteric site prevents peptide binding at the active site [17]. From the above discussion, it is clear that the discovery of new allosteric sites may generate opportunities for pharmaceutical development and increases the understanding of basic biological processes.

In our previous study, PH1704 was observed to be an allosteric enzyme through experimental methods [18,19]. Anion allosteric regulation showed that Cl^−^ functions as an allosteric inhibitor [18,19], but we still do not know how Cl^−^ stabilizes distinct contact species and controls the enzyme activity. In this study, quantum mechanical calculations were employed to determine the binding mode of Cl^−^. A molecular dynamics (MD) method was used to explore the allosteric regulation. Our findings revealed that at least two processes are involved in functionally coupling the allosteric site and the active center of PH1704, that is: (*i*) Cl^−^ binding, a process that entails masking the conformational stabilization of the subunit contact, is not beneficial to enzyme activity; (*ii*) stabilization of the active conformation of the common H bond between His101 and Glu474, may be caused by the unoccupied site at the two contacts of Cl^−^. This H bond enhances the rate of formation of the active conformer. Therefore, further experimental and theoretical studies for PH1704 are necessary.

## 2. Results and Discussion

### 2.1. Quantum Mechanical Calculation to Determine the Cl^−^ Binding Mode

Motif Scan [20] was used to search for the Cl^−^ binding site. It can be found that there was a possible amidation site (refering to the amidation reaction that acylates the NH group of an amino acid with oxygen, chloride, and sulfur atoms) in Arg113 and Lys116. To explore the Cl^−^ binding mode, we checked the crystal structure of PH1704 carefully. Two SO_4_^2−^ were bound in the AC contacts: Arg113 formed two salt bridges with the SO_4_^2−^ O atom and Asn129 formed a hydrogen bond with SO_4_^2−^ through Wat744 (Figure 1b). Arg113 and Asn129 may come in with an ion. Hence, we proposed that Cl^−^ may bind at the same site as SO_4_^2−^: to form a salt bridge with Arg113 and form a hydrogen bond with Asn129 through water.

Rosetta design can be used to redesign an existing protein for increasing binding affinity. We used Rosetta design to get best theoretical mutant, R113T. As for the experimental data, the negative controlling effect disappeared for the R113T mutant for substrate R-AMC, and the apparent *k*_cat_/*K_m_* value was 4.4-fold higher than that of PH1704 for substrate R-AMC, indicating that it became a standard Michaelis–Menten enzyme [18,19].

Based on the above data, Arg113 may be involved in the allosteric regulation. However, the exact mechanism of the binding mode with the allosteric inhibitor Cl^−^ is still unknown. Sheng *et al.* were the first to observe that structural stabilization at the active site caused by the binding of an anionic allosteric activator [21]. Structural and biochemical data showed that mutations of some residues at this site influenced the binding of SO_4_^2−^ and affected the enzymatic activity. In the present study, we find another anionic allosteric inhibitor, namely, Cl^−^.

The crystal structures of L-arginine·2HCl·H_2_O have been determined [22], but some basic questions relate to PH1704 remain unanswered. An example, one of these questions is: “should Asn129 also form a hydrogen bond with Cl^−^ through water?” Detailed understanding of the Cl^−^ binding mode at the atomistic level is necessary for further successful rational design of PH1704.

**Figure 2:**
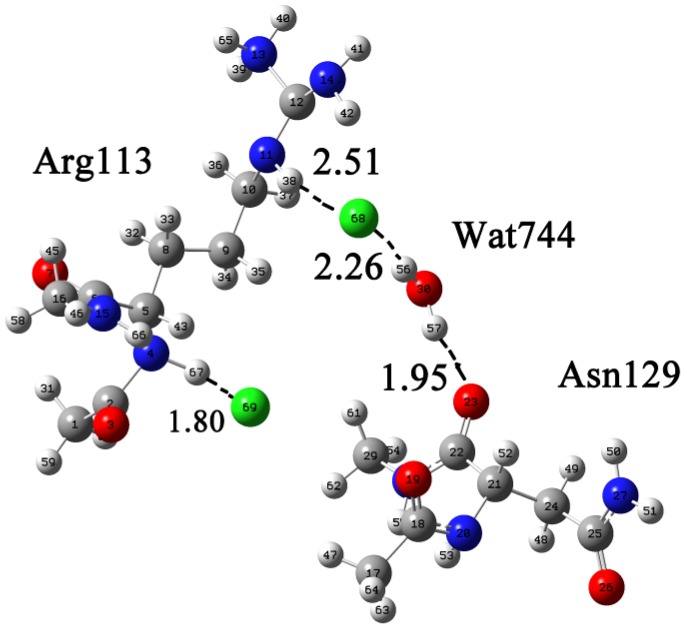
The calculated Arg∙2HCl∙H_2_O∙Asn supermolecular system displayed in Gaussian View. The coordinates of Arg113, Asn129, and Wat744 were taken from Protein Data Bank [23], and two Cl^−^ and hydrogen atoms were manually added by Gaussian View.

Figure 2 shows the numbering of the atoms of Arg113 and the binding mode of Cl^−^. The conclusion about the formation of a neutral carboxyl group, and hence double charged Arg^2+^ cation, was made on the basis of bond lengths C=O (1.22 Å) and C–OH (1.32 Å). An intermediate region was found, where the strongest van der Waals and the weakest hydrogen bonds coexist. For the N–H (38)···Cl (68) and O–H(56)···Cl (68) contacts, the regions are located at 2.51 Å and 2.26 Å, respectively. The corresponding N–H (67)···Cl (69) distance (1.81 Å) fits to intermediate region.

**Figure 3:**
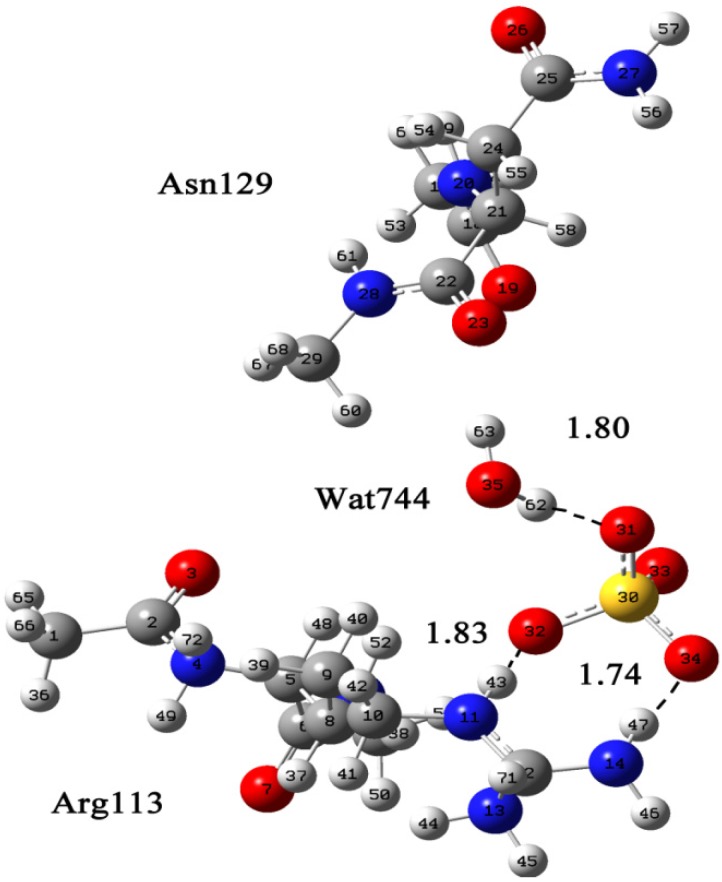
The calculated Arg∙SO_4_^2^∙H_2_O∙Asn supermolecular system displayed in Gaussian View. Arg113, Asn129, Wat744, and one SO_4_^2−^. The coordinates of Arg113, Asn129, Wat744, and one SO_4_^2−^ were taken from Protein Data Bank [23].

Determining the state of water molecule in the structure is important. In this system, a proton donor water molecule formed hydrogen bonds with Cl^−^ (68) and Asn129. Figure 3 shows the numbering of Arg113 and the binding mode of SO_4_^2−^. As shown in Figure 3, an intermediate region of coexistence of the strongest van der Waals and the weakest hydrogen bonds is found. For N (11)–H (43)···O (32) and N (14)–H (47)···O (34) contacts, the regions are located at 1.83 Å and 1.74 Å, respectively. Subtle differences were seen between models A and B: two Cl^−^ formed two hydrogen bonds with Arg113 head and tail, whereas SO_4_^2−^ only formed one hydrogen bond with the guanidine group of Arg113, so it can be concluded that Arg113 and Asn129 are coordinated with the allosteric inhibitor, Cl^−^. Thence Arg113 is involved in allosterc regulation, which is consistent with the experimental data. Asn129 may also take part in the allosteric action as it forms a hydrogen bond with Cl^−^ through water.

### 2.2. Protein-Substrate Complex Preparation

The docking scores between L-arginyl-7-amido-4-methylcoumarin (R-AMC) and PH1704 with AutoDock vina, AutoDock 4.2 and Dock 6.6 are listed in Table 1. The docking score from AutoDock vina was lower than with the other software, so the docked complex from AutoDock vina was chosen for further study.

**Table 1:** The docking scores between R-AMC and PH1704 calculated with AutoDock vina, AutoDock 4.2 and Dock 6.6.

Substrate/PH1704	AutoDock vina	AutoDock 4.2	Dock 6.6.
R-AMC	−7.17	−6.65	−6.55

**Figure 4:**
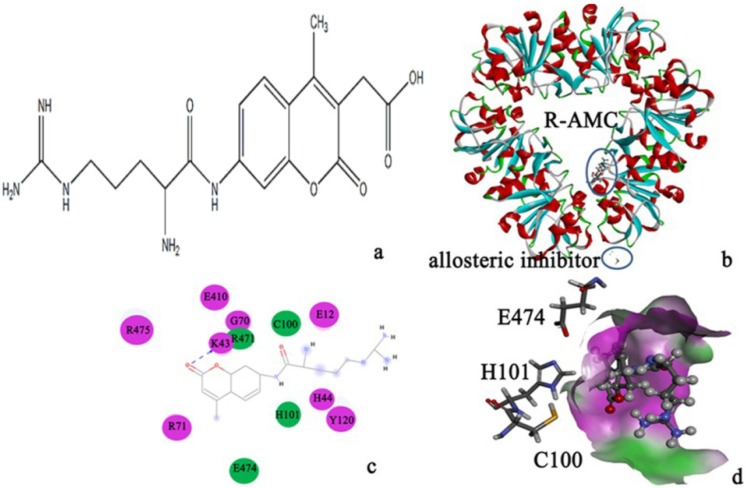
(**a**) R-AMC; (**b**) The docked complex; R-AMC located in the AC contacts; (**c**) The important residues in R-AMC binding calculated by Discovery studio 3.5 client. Color green represents for van der Waals contacts with R-AMC, and color purple represents for electrostatic contacts with R-AMC; (**d**) The surface around the R-AMC generated by Discovery studio 3.5 client. R-AMC is in the active pocket and near the active triad (C100, H101 and E474).

The substrate, R-AMC (Figure 4a), was docked to PH1704 in AC contacts. As seen from Figure 4b, we can conclude that R-AMC located in the AC contacts away from the allosteric site (Arg113 and Ans129). There were 12 residues (Arg475, Glu410, Gly70, Lys43, Arg471, Cys100, Glu12, Arg71, His101, His44, Tyr120, and Glu474) around R-AMC (Figure 4c), and the results were consistent with those obtained by Du and Zhan [1,2] (Figure 4d), and it is easy for the reaction to occur, so the PH1704–R-AMC complex can be used for further study.

### 2.3. Molecular Dynamics Simulations to Study Allosteric Regulation by Cl^−^

Chloride anions have been reported to function as organic guests for surpermolecular systems [24]. However, the present study is the first to observe that Cl^−^ produces appreciable changes in the catalytic activity site of the protease when used as an allosteric inhibitor.

An important goal for studying the allosteric enzyme PH1704 is to regulate the activity of catalysis through the addition of small molecules that change the enzyme structure of the catalyst and in turn control catalytic reaction rates and product distributions. We aim to determine the regulation induced by the binding of Cl^−^ on the function, structure, and flexibility of PH1704.

**Figure 5:**
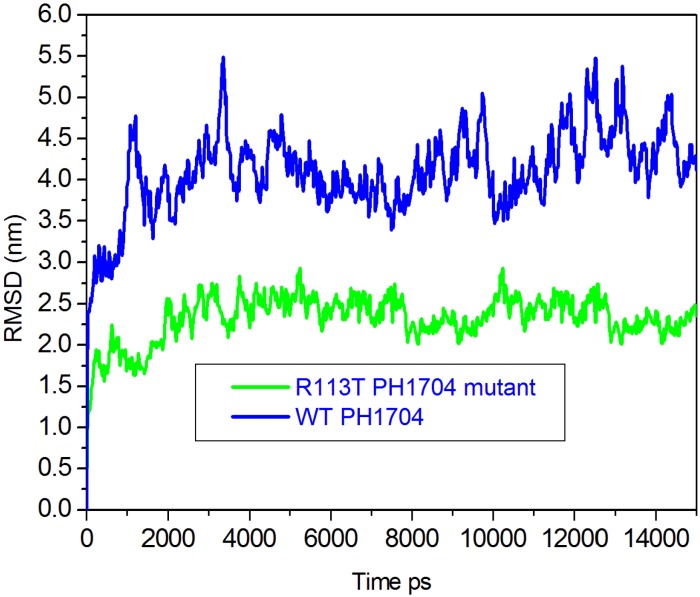
Plot of the RMSD that is experienced by PH1704 over the course of the trajectories for PH1704–R-AMC of Cl^−^ bind state (blue), and R113T–R-AMC mutant (green).

To obtain a deeper understanding of the structural and dynamic basis for the allosteric effects in PH1704, 10 ns explicit-solvent molecular dynamics simulations were used. In this study, two separate simulations were conducted in each of the aforementioned cases, with PH1704–R-AMC of the Cl^−^-binding state and the R113T–R-AMC mutant. Extensive analysis of conformational change and motion was conducted through the computation of the root-mean-square deviation (RMSD), order parameters, dynamical cross-correlation maps, and essential dynamics. Figure 5 shows the RMSD of *C*α atoms with respect to their initial positions. Sharp increases in the R113T–R-AMC mutant were observed from the plot during the first 4,000 ps. The average RMSD was below 2.5 Å over the entire simulation for the complex. However, the simulation trajectories of the PH1704–R-AMC of the Cl-bind state appeared to be poorly equilibrated, with an average RMSD value of 4.0 Å over the last 11,000 ps, indicating that the structure of the R113T–R-AMC mutant was stable during data collection.

**Figure 6:**
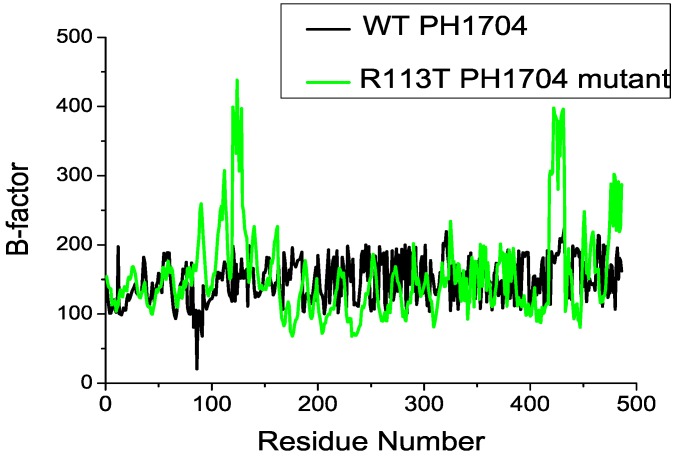
Backbone residue-based B-factors calculated over the 15 ns time window for PH1704 (black), and R113T mutant (green).

The calculation of B-factors provides insight on the identification of the rigid and flexible parts of a protein. However, comparing them in a qualitative manner through the simulation model is still beneficial. Figure 6 shows the B-factors for *C*α atoms calculated from the MD simulation of PH1704–R-AMC complex.

**Figure 7:**
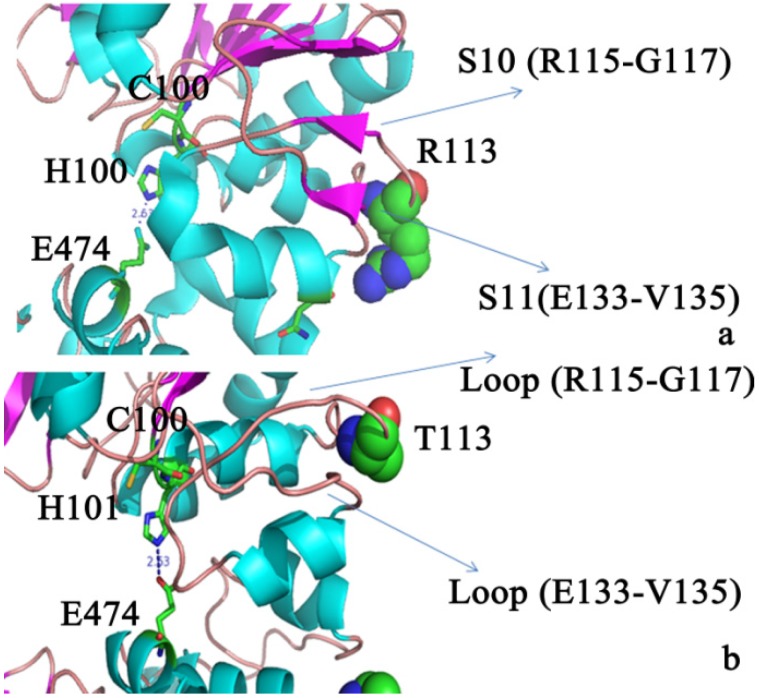
(**a**) Conformation change in two contacts in the PH1704; (**b**) In the R113T mutant, S10 and S11 became a loop.

As shown in this figure, simulation of B-factors often (although not always) peak for the same residues. Moreover, residues around or right at the turns or loops are more flexible on the basis of these peaks, which is consistent with the fact that these sites fall short of the stable hydrogen bond network. The secondary structures were well maintained. In all the systems, the sheets of the positions of S10 (residues 115–117) and S11 (residues 133–135) in the A and C contacts were lost and formed a loop (Figure 7). The most flexible regions corresponded to the AC contact (residues 115–135) in the R113T mutant. Large mobility of these domains is consistent with the need for this region to undergo conformational changes in oligomeric association of the AC subunit. The above result indicates that Cl^−^ binding was beneficial for the main rigid region of the two contacts. Nevertheless, the flexibility of the two contacts was beneficial for the enzyme activity.

**Figure 8:**
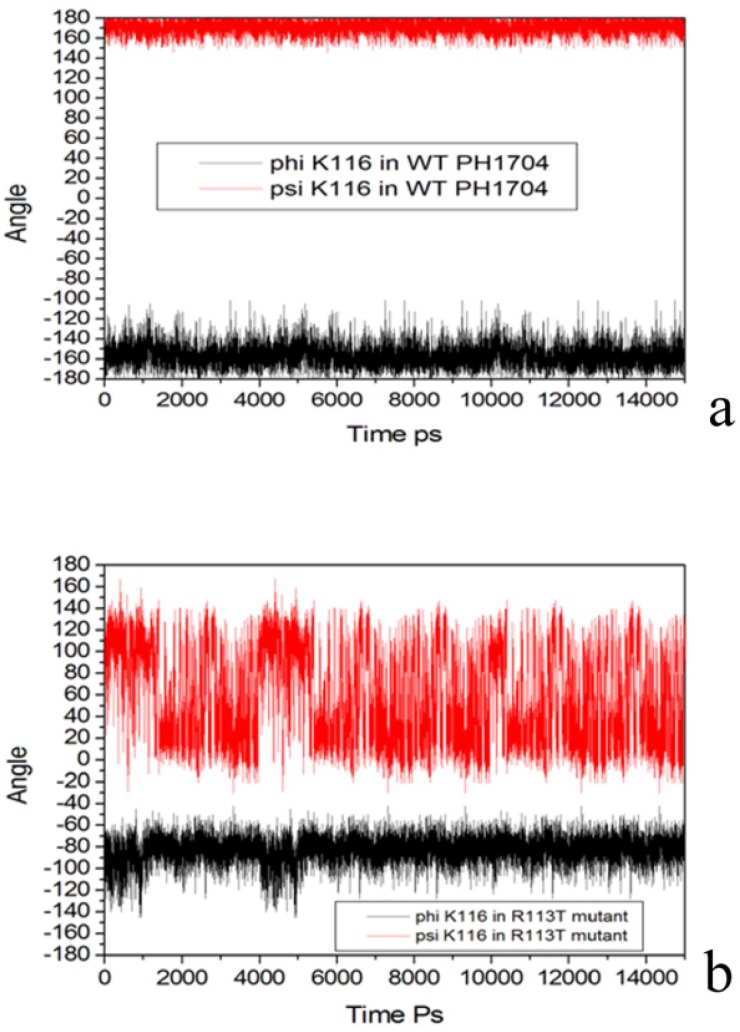
(**a**) Phi and psi dihedral angles of Lys116 in R113T mutant; (**b**) Phi and psi dihedral angles of Lys116 of Cl^−^ bind state during MD.

To investigate its flexibility, the phi and psi dihedral angles were plotted against MD time as shown in Figure 8a.

The variations of the phi and psi dihedral angles were placed within 60 degrees for Lys116 in the WT PH1704. However, for the R113T mutant state, the phi and psi dihedral angles were positioned at 160 degrees (Figure 8b). As a result, the difference between Lys116 in the WT PH1704 included in the S10 and Lys116 in the R113T mutant, which were not included in the sheet, can cause changes in the stability of the AC contact.

**Figure 9:**
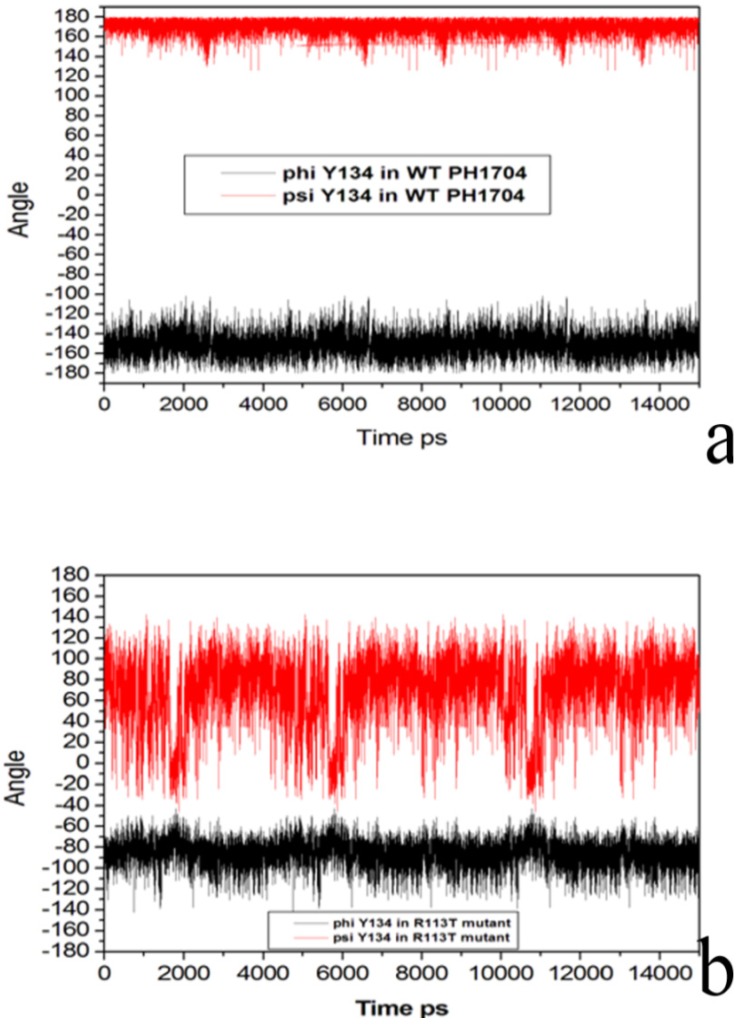
(**a**) Phi and psi dihedral angles of Tyr134 in R113T mutant; (**b**) Phi and psi dihedral angles of Tyr134 of Cl^−^ bind state during MD.

As shown Figure 9a, the variations of phi and psi dihedral angles were placed within 60 degrees for Tyr134 in the WT PH1704. However, the phi and psi dihedral angles for the R113T mutant were positioned at 180 degrees (Figure 9b). The results above suggest that the R113T mutant is more flexible in the AC contacts, and is beneficial to the reaction.

Hydrogen bonding (salt bridge) was present between His101 and Glu474, Ser108 and Asp525 and Arg477 and Asp126 (listed in Table 2). For the R113T mutant, S10 and S11 became a loop (Figure 6). S10 and S11 were near the H7 helix (residues 121–127) which was close to the AC contacts. This conformational difference appeared to be correlated to the binding of the Cl^−^ ion at the allosteric site and had an important role in the regulation of enzymatic activity.

**Table 2:** The interatomic contacts between A and C subunit.

A subunit	C subunit	Distance (Å)
S108 OH	D525 COO^−^	2.64
H101 NH	E474 COO^−^	1.66
D126 COO^−^	R477 NH^+^	2.08

The hydrogen bond (salt bridge) has an important function in this system [25]. Therefore, the hydrogen bonds in the three mutants were monitored. Table 3 summarizes this noncovalent interaction in the AC contact occupancies at the last 11 ns. The hydrogen bonds between the AC contacts in the WT enzyme–substrate complex simulation, contained Asp525 and Ser108, and His101 and Glu474. The Ser108–Asp525 in the R113T mutant formed a hydrogen bond with occupancies of 97.35%. While the residue His101 formed one hydrogen bond with Glu474 with occupancies of 90.25% in the R113T mutant. Therefore, the hydrogen bond in the mutant was stable, but the hydrogen bond of Ser108–Asp525 decreased from 97.35% to 49.43% in WT type. The hydrogen bond of His101–Glu474 in WT type (42.71% occupancy) also decreased. The salt bridge of Arg477 and Asp126 (32.12% in the WT type) in the R113T mutant increased. In a word, in the R113T enzyme–substrate complex, all the hydrogen bonds and salt bridges were stable, but in the relationships fluctuated the WT enzyme–substrate during MD. When the ion pair between the AC contacts increases, the dimer may be compact, thereby benefiting the catalytic base hydrogen bond (His101 and Glu474). These results may partially explain why the former mutation resulted in the enhancement of the catalytic efficiency of the PH1704.

**Table 3:** Hydrogen bond (salt bridge) contacts between the WT PH1704 and the R113T mutant during the MD phase.

Residue	Amino acid group	Residue	Amino acid group	Occupancy (%)	
				R113T	WT
A S108	OH	C D525	COO^−^	97.35	49.43
A H101	NH	C E474	COO^−^	90.25	42.71
A D126	COO^-^	C R477	NH^+^	72.43	32.12

The pKa values were calculated for all ionizable residues of PH1704 using the program H++3.0 [26]. The calculations were performed for the WT type and R113T mutant. The catalytic activity of Cys100 was believed to participate in the shuttling of protons from His101. His101 showed that the pKa values significantly shifted from the standard values. The pKa of His101 shifted from 7.0 units in the WT type to 11.16 units in the R113T mutant. Based on the crystallographic data His101 is involved in interactions with the catalytic residue Cys100 (Figure 1a). His101 forms a hydrogen bond with Glu474 to function as a catalytic base. In the active site, His101 in the WT type has lower pKa than the R113T mutant. The catalytic efficiency of PH1704 is related to the presence of the acid⁄base catalyst (His101–Glu474) of PH1704, and therefore to the pKa value of His101. The higher the pKa value of His101, the stronger the proton transfer between His101 and Cys100, and the stronger resulting in catalytic reaction becomes.

In summary, when the Arg113 mutant becomes Thr, Cl^‒^ is not located in the AC contacts and two β sheets (S10 and S11) become loops. This flexible domain may increase the formation of the hydrogen bonds between the AC contacts including His101–Glu474 and His501–Glu74, which function as catalytic bases.

**Figure 10:**
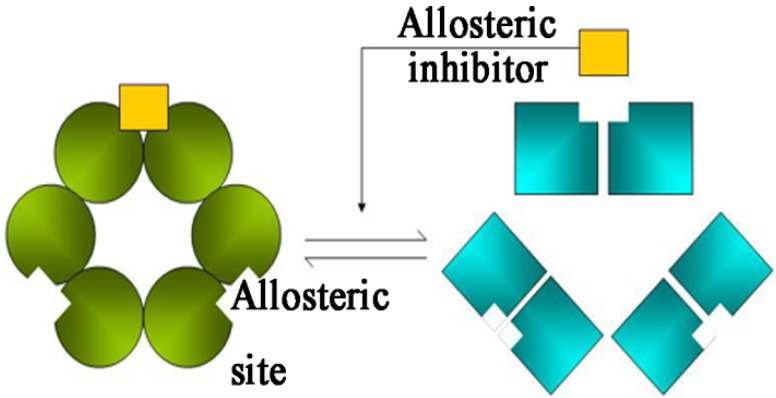
Cooperativity: a cartoon representation of the Monod–Wyman–Changeux model of allosteric transitions. Three binding sites for 12 Cl^−^ and three pairs of catalytic triads in PH1704 are observed. The activity of PH1704 is directly under allosteric control via the bound Cl^−^.

Figure 10 shows the cooperativity, which is a schematic representation the Monod–Wyman–Changeux model of allosteric transitions [27]. A symmetric, multimeric protein can exist in one of two different conformational states, namely, active and inactive conformations. Each subunit has a binding site for an allosteric inhibitor and an active or binding site. PH1704 has three binding sites for 12 Cl^−^ and three pairs of catalytic triads. When Cl^−^ is not located in the enzyme, the two contacts become flexible, thereby helping increase the stability of the enzyme–substrate complex. Thus, the activity of PH1704 is directly under allosteric control via the bound Cl^−^ (allosteric inhibitor).

## 3. Experimental

### 3.1. Quantum Mechanical Calculation Method

All calculations were performed with the Dmol^3^ module in Material Studio program package [28]. In this system, C, N, and O atoms were fixed. Based on the optimized structure, the single point energy of all species was obtained by GGA/BLYP/DNP with the basis set 6-31+G (d) with the Gaussian 03 package [29,30,31]. Two quantum chemical models, ranging from 69 atoms to 72 atoms, were used. The following groups were included: (1) Model A composed of Arg113, Asn129, Wat744, and two Cl^−^; (2) Model B composed of Arg113, Asn129, Wat744, and one SO_4_^2−^. The coordinates of Arg113, Asn129, Wat744, and one SO_4_^2−^ were taken from Protein Data Bank [23], and two Cl^–^ and hydrogen atoms were manually added by Gaussian View [32].

### 3.2. Protein-Substrate Complex Preparation

The 3D structure of PH1704 was downloaded from Protein Data Bank (PDB id: 1G2I). Water molecules and other heteroatoms were removed, and the program PDB2PQR 1.8 [33] was used to assign position-optimized hydrogen atoms, utilizing the additional H++ 3.0 [26] algorithm with a pH of 7.5 to predict protonation states. The 3D structure of the substrate (R-AMC) was built by InsightII/Builder program and was further optimized using the 6-31G (d) set with the Gaussian 03 package [29,30,31]. The R113T mutant was made by Pymol. The six subunits of PH1704 contained 966 residues were used for further docking and MD study. The substrate R-AMC, was docked in the AC contacts which contained catalytic triad: Cys100, His101 and Glu474 and the allosteric site: Arg113 and Asn129 in A subunit. Two Cl^–^ and one water were manually added by Gaussian View [32] in the allosterc site according to Quantum mechanical calculation results.

Autodock Vina, AutoDock 4.2 and Dock 6.6 were used for perform docking [34,35,36]. The grid size for Autodock Vina and AutoDock 4.2 docking was 56 Å × 56 Å × 56 Å. The created clusters were enclosed in a box, and force fields scoring grids were generated by DOCK 6.6 [36]. The maximum number or orientations of the ligand was limited to 5000, and only the 20 lowest solutions were saved and evaluated.

### 3.3. Molecular Dynamics Simulations

Amber 10.0. [37] was used for MD simulation. For the ligand, R-AMC, GAFF force field [38] parameters and RESP partial charges [39] were assigned using the ANTECH Amber program implemented in Amber 10.0. Several sets of MD simulations were carried out on the protein–ligand complex and the mutant structures using the Amber 10.0 simulation package and the Parm06 force field [37], respectively. The program LEaP was used to neutralize the complexes. The SHAKE algorithm [40] was used to constrain the bonds with hydrogen atoms. The complexes were solvated in an octahedral box of water, with the shortest distance between any protein atom and the edge of the box being approximately 10 Å. The particle mesh Ewald (PME) method [41] was employed to calculate long-range electrostatic interactions. Then the complexes were minimized for 1,000 steps with the steepest descent method using the PME MD module of Amber 10.0. The systems were equilibrated at 353 K for 50 ps. MD simulation was employed to record time trajectory after 50 ps equilibration. The two systems of the complex were simulated for 15 ns. The time step used in all calculations was 2.0 fs. Coordinates were saved every 1 ps for subsequent analysis.

## 4. Conclusions

The intracellular protease from *P. horikoshii* (PH1704) is the first allosteric enzyme that has negative cooperativity with chloride ion. Quantum mechanical calculation identified the binding mode of Cl^−^ with Arg113 and Asn129. Arg113 may be involved in the allosteric mechanism because it forms a salt bridge with two Cl^−^. The molecular dynamics simulation was used to investigate the allosteric mechanism of PH1704. Our findings indicated that at least two components are involved in functionally coupling the allosteric site and the active center of PH1704, namely: (*i*) Cl^−^ binding process that masks the conformational stabilization of the subunit contact, is not beneficial to enzyme activity; (*ii*) stabilization of the active conformation of the H bond between His101 and Glu474, may be caused by the unoccupied site at the two contacts of Cl^−^. Thus, further experimental and theoretical studies are necessary.
